# Tarlov cysts and sexual dysfunction: A multidisciplinary approach to evaluation and surgical treatment

**DOI:** 10.1016/j.xnsj.2026.100866

**Published:** 2026-02-13

**Authors:** Shashank Coorapati, Irwin Goldstein, Sue W. Goldstein, Barry R. Komisaruk, Choll W. Kim

**Affiliations:** a University of California, Riverside School of Medicine, Riverside, California, United States; b Excel Spine Center, San Diego, California, United States; c San Diego Sexual Medicine, San Diego, California, United States; d University of California San Diego Health, East Campus, La Jolla, California, United States; e Department of Psychology, Rutgers University, Piscataway, New Jersey, United States

**Keywords:** Symptomatic Tarlov cysts, Pelvic pain, Urinary dysfunction, Sexual dysfunction, Persistent genital arousal disorder, Genito-pelvic dysesthesia, Perineural cysts, Sacral radiculopathy, Tarlov cyst surgery, Dural ectasia

## Abstract

**Background:**

Persistent Genital Arousal Disorder/Genito-Pelvic Dysesthesia (PGAD/GPD) involves chronic, unrelenting arousal and dysesthesia of the genito-pelvic region. In 2012, Komisaruk and Lee identified a high incidence of sacral Tarlov cysts in patients with PGAD/GPD, prompting development of a multidisciplinary diagnostic and treatment algorithm. This study reviews surgical outcomes for patients with PGAD/GPD in whom a Tarlov cyst was identified as the symptomatic driver.

**Methods:**

A retrospective review of prospectively collected data was conducted on patients with PGAD/GPD who underwent Tarlov cyst excision and imbrication from 2017 to 2023. The diagnostic algorithm included neurogenital testing, sacral MRI, and assessment for pelvic or end-organ triggers.

When one or more cysts were suspected as the etiology, a targeted diagnostic injection with anesthetic and steroid confirmed the diagnosis.

Operative reports, imaging, and pre/postoperative clinic visits were reviewed to assess complications, readmissions, reoperations, and symptom changes. Outcomes were measured using the Patient Global Impression of Improvement (PGI-I), Oswestry Disability Index (ODI), Sexual Distress Scale-Revised (SDS-R), and Visual Analog Scale (VAS) for back pain.

**Results:**

Nineteen patients (mean age 45.5 ± 15.9 years) with an average follow-up of 18.2 ± 10.5 months were included. Fourteen patients (73.7%) reported postoperative improvement on the PGI-I: 2 “very much better,” 9 “much better,” 3 “little better,” and 5 with no improvement. No patients reported worsening symptoms. All were discharged the same day, with no cerebrospinal fluid leaks, wound issues, or cyst recurrences. Oswestry Disability Index improved from 32.9 to 28.4, SDS-R from 30.9 to 26.8, and Visual Analog Scale back pain from 4.8 to 3.2.

**Conclusions:**

Symptomatic Tarlov cysts can contribute to PGAD/GPD and associated sexual dysfunction. Early detection with a structured diagnostic algorithm support accurate patient selection and safe, effective surgical treatment, offering meaningful symptom relief for appropriately selected patients.

## Background

Tarlov cysts are cerebrospinal fluid-filled dilatations that develop in the perineural space between the perineurium and endoneurium, typically at the junction of the posterior nerve root and the dorsal root ganglion. These cysts, classified as Type II spinal meningeal cysts and/or perineural cysts, maintain communication with the subarachnoid space. Nerve fibers are located within the cyst or along its walls. Tarlov cysts are most commonly found in the sacral roots but can occur at any spinal level [1].

The etiology underlying Tarlov cysts includes congenital, inflammatory, and traumatic factors [2]. The mean global pooled prevalence of Tarlov cysts is estimated to be 4.27% [3].

The natural history of symptomatic Tarlov cysts often involves progressive enlargement over time due to the increased hydrostatic pressure of cerebrospinal fluid (CSF). A one-way valve-like mechanism permits CSF influx while restricting efflux, leading to cyst expansion. This growth can stretch and/or compress the nerve root filaments within the cyst, leading to a variety of sensory and motor disturbances [4,5]. While the majority of cysts remain asymptomatic, approximately 20% may present with symptoms [6]. Symptomatic Tarlov cysts have historically been associated with a wide range of clinical manifestations that affect the organs of the genito-pelvic region, such as bladder incontinence, sexual dysfunction, and Genito-Pelvic Dysesthesia (GPD), including pelvic pain and Persistent Genital Arousal Disorder (PGAD) [7].

The present study followed patients with Tarlov cyst-induced PGAD/GPD, a debilitating condition characterized by chronic, unrelenting arousal and dysesthesia symptoms in the genito-pelvic region [8]. Research on the association between Tarlov cysts and sexual dysfunction remains limited. One cohort study of 11 patients with PGAD that underwent microsurgical treatment for Tarlov cysts reported that 91% of patients experienced complete resolution or significant reduction of PGAD symptoms following surgical treatment [9]. Another study involving 65 female patients with Tarlov cysts concluded that the cysts may have a clinically significant impact on urogenital function as evidenced by symptoms of urinary urgency and urodynamic findings associated with urgency [10].

The present report demonstrates the utility of a multidisciplinary management algorithm for identifying a subgroup of patients with PGAD/GPD and Tarlov cysts, and of a treatment strategy supported by the outcomes of patients who underwent an excision and imbrication procedure for symptomatic Tarlov cysts. To our knowledge, this is the largest series of such patients reported to date.

## Materials and methods

### Study design

Following Institutional Review Board approval, a retrospective cohort analysis was performed using prospectively collected data from 2017 to 2023 on the charts of patients diagnosed with Persistent Genital Arousal Disorder/Genito-Pelvic Dysesthesia (PGAD/GPD) who underwent a Tarlov cyst imbrication and excision. This multidisciplinary approach involved specialists in sexual medicine, urology, neurophysiology, and spine surgery. Patients were selected using a structured management algorithm that focused on those with concurrent Tarlov cysts who experienced persistent, distressing GPD for a duration of at least 3 months. Only patients with a minimum of 6 months' postoperative follow-up were included in the analysis.

Patient-reported outcome measures (PROMs) were systematically collected at each follow-up visit, including the Patient Global Impression of Improvement (PGI-I), the Visual Analog Scale (VAS) for back pain, the Sexual Distress Scale-Revised (SDS-R), and the Oswestry Disability Index (ODI). Success of the surgical intervention was primarily measured through the PGI-I, a validated and widely accepted patient-reported outcome measures that utilizes a 7-point Likert scale to gauge the subjective improvement of patients following treatment. In addition, the preoperative baseline scores of the other validated instruments were compared to the most recent postoperative follow-up to quantify overall treatment efficacy.

### Variables

Demographic and surgical variables recorded in this study included patient age, sex, primary and secondary spinal pathologies, and the specific vertebral levels where cyst excision and imbrication procedures were performed. Surgical outcomes were assessed based on length of stay (LOS), total operative time (cut-to-close), perioperative complications, readmissions, reoperations, and any new or worsening neurological symptoms.

### Eligibility criteria

Patients were evaluated through a multidisciplinary approach. The first step involved a comprehensive evaluation by a sexual medicine expert to identify patients who were diagnosed with PGAD/GPD and underwent a thorough evaluation including neurogenital testing and regional anesthesia testing.

Neurogenital testing consisted of quantitative sensory testing (QST) for warmth, cold and vibration, sacral dermatome testing, and bulbocavernosus muscle reflex (BCR) latency testing as previously described [7,8]. Briefly, patients with PGAD/GPD in the study cohort who had abnormal findings on all the neurogenital tests were considered to have a pattern consistent with sacral radiculopathy.

Patients with abnormal QST and BCR latency test results but normal sacral dermatome findings were considered to have a pattern consistent with PGAD/GPD from pudendal neuropathy and not sacral radiculopathy.

Patients with abnormal QST and abnormal sacral dermatome but normal BCR latency test results were considered to have a pattern consistent with PGAD/GPD pathology above the conus medullaris, including upper spinal cord and/or brain, and not sacral radiculopathy.

Regional anesthesia testing included vestibular anesthesia testing (VAT) or pudendal nerve blocks, to test for possible PGAD/GPD triggers in region 1 (end organ) or region 2 (pelvis/perineum), respectively, as described previously [7,8]. Other regions include region 4 (spinal cord) and region 5 (brain). In addition, lumbosacral MRIs were evaluated for the presence of Tarlov cysts.

To further refine the diagnostic approach, patients whose evaluations indicated abnormalities in region 3 (lumbosacral spine) were referred to a pain medicine specialist for a diagnostic caudal epidural steroid injection containing lidocaine or bupivacaine [[12], [13]]. This injection aimed to determine whether administration of a local anesthetic agent would result in significant clinical reduction of PGAD/GPD symptoms within the first 4 hours of the injection.

Patients with confirmed PGAD/GPD localized to the lumbosacral region who experienced a positive diagnostic response then underwent surgery via Tarlov cyst excision and imbrication.

The multidisciplinary diagnostic and treatment algorithm used in this study has been previously described in detail, including a visual schematic, in prior published work [8]. The present study applies this established framework to a surgical cohort with sexual and pelvic dysfunction associated with Tarlov cysts.

### Radiographic evaluation

**Fig. 1:**
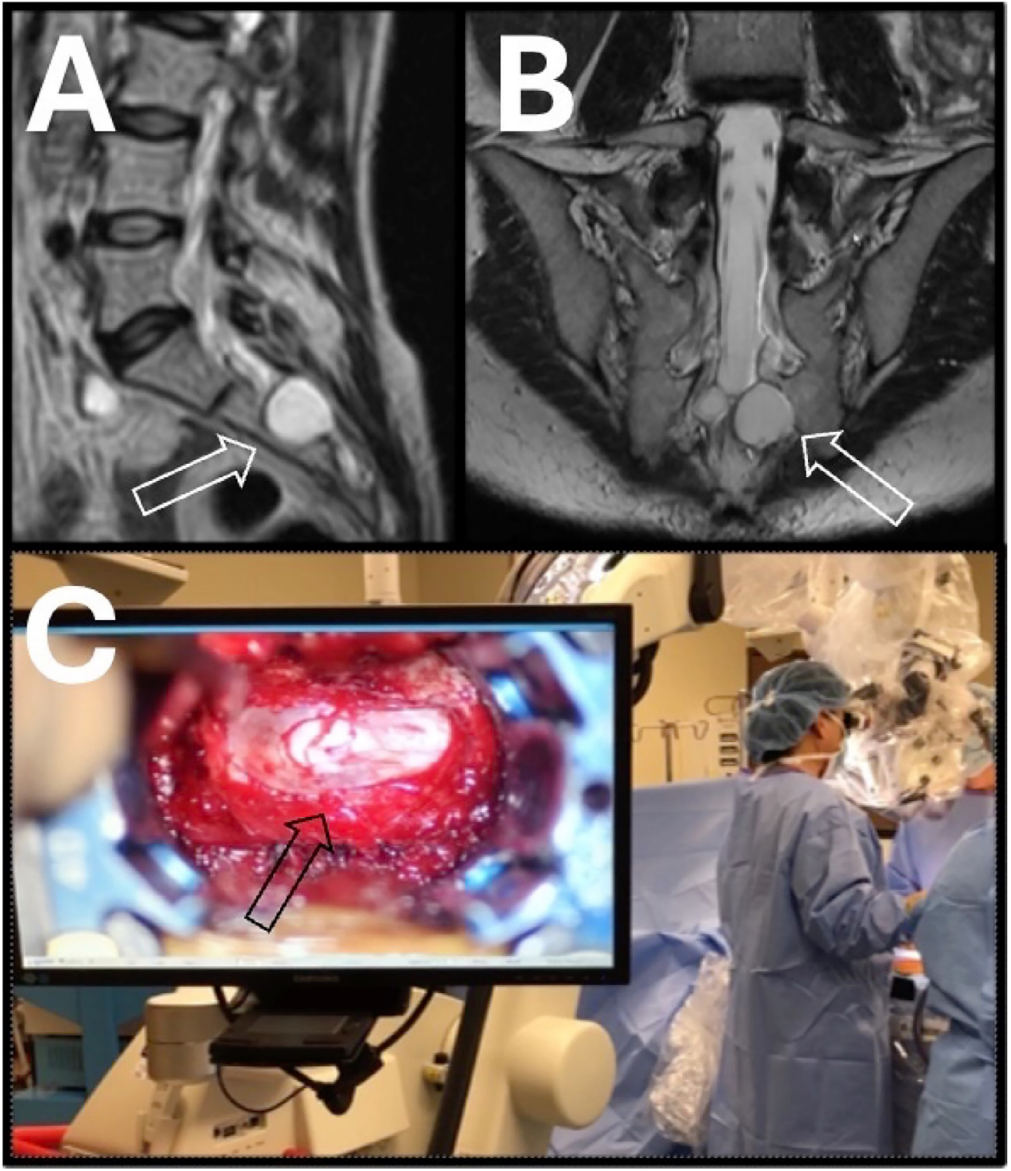
T2 MR image in the sagittal (A) and coronal (B) planes of a typical Tarlov cyst. When possible, the images are obtained orthogonal to the sacrum to obtain the most comprehensive view of the cyst shape, size, and location. The left S3 Tarlov cyst causes bony erosion (open white arrow). (C) Intraoperative picture with view of microscope monitor showing the gross appearance of the Tarlov cyst. Increased vascular injection, consistent with inflammation, can be appreciated (black arrow).

All patients were evaluated with MRI. Most patients had routine lumbar spine MRI. Some patients underwent specialized imaging to optimize visualization of the sacral Tarlov cysts. This “Tarlov cyst MRI protocol” consisted of slices oriented to obtain true axial, sagittal, and coronal images of the sacrum (Fig. 1).

### Diagnostic injections

Targeted diagnostic injections utilized a standard caudal epidural injection with anesthetic (lidocaine or bupivacaine) to assess for temporary PGAD/GPD symptom reduction. Patients who reported a reduction of more than 50% in symptoms and/or a postinjection PGI-I score of 1, 2, or 3 were considered to have a “positive” diagnostic response. One patient underwent surgery despite a negative diagnostic response and 1 patient underwent surgery without diagnostic injections.

### Surgical technique

All patients underwent surgery via excision and imbrication, as previously described [11]. All patients underwent surgery using image guidance/navigation using the Stealth O-Arm System (Medtronic, Littleton, MA). Briefly, patients were placed in the prone position, and the posterior superior iliac crest was used for the Stealth navigation patient reference pin.

A midline skin incision was used to perform a subperiosteal dissection of the sacral lamina. All dissection was performed under the operating microscope. Sacral laminectomies were then performed to expose the entirety of the Tarlov cyst(s). The normal boundaries of the cyst were identified and a stay suture placed to control the dural membrane.

A sharp 11 blade scalpel was used to incise the dura over a region free of nerves. The intrathecal space was then entered and the cavity explored with a fine probe. Nerve rootlets were carefully identified.

**Fig. 2:**
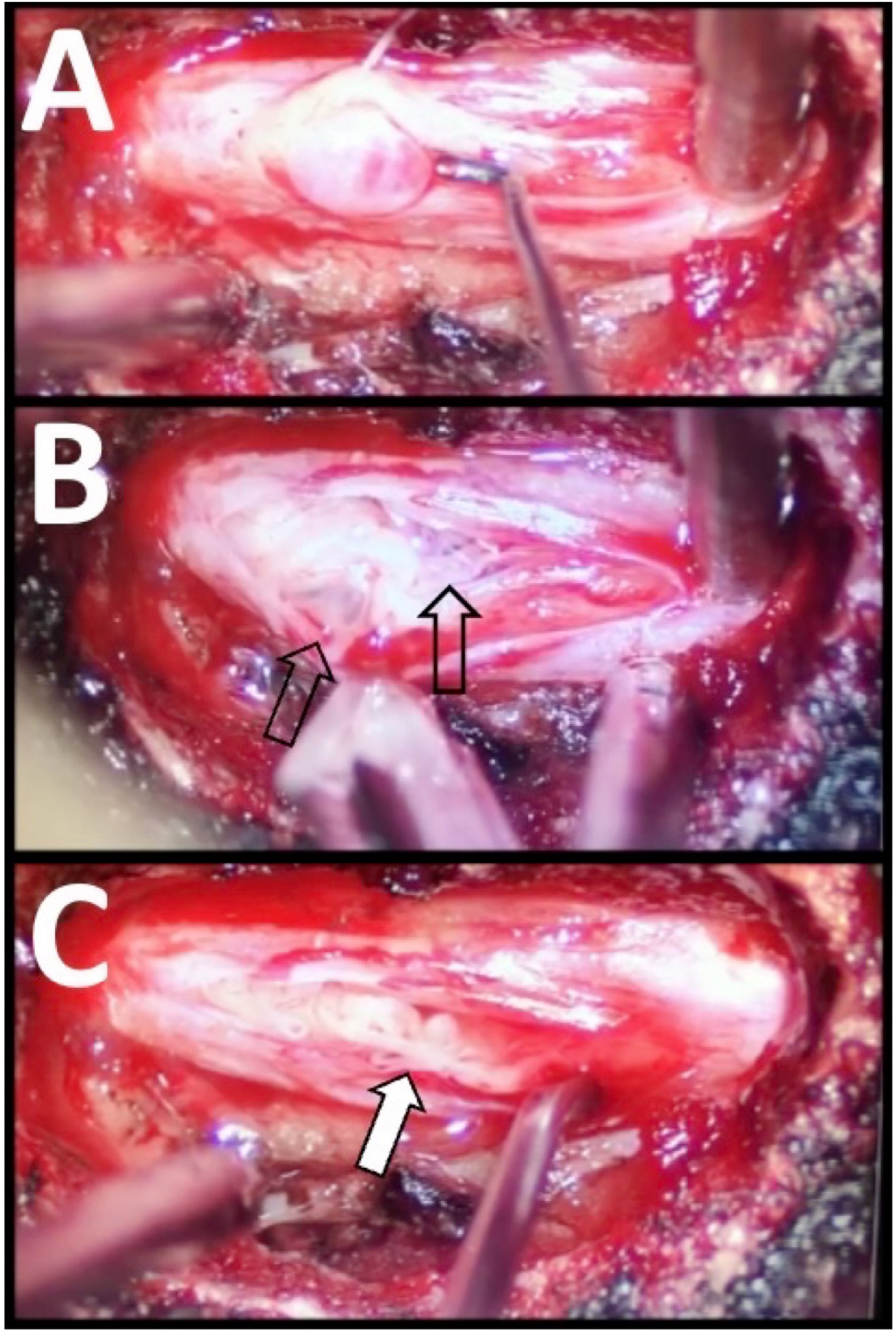
Intraoperative microphotographs of a Tarlov cyst after dural incision showing an arachnoid bleb containing cerebrospinal fluid immediately adjacent to the tip of the nerve hook (A). Upon entering the intradural space, the trabeculae that suspend and support the nerve fibers within the cerebrospinal fluid-filled cavity of the Tarlov cyst are hypertrophic (B). They appear to distort that path of the nerve fibers and often contain distinct pockets of trapped CSF (black arrow). This may lead to obstructions that trap CSF and alter the path of the nerve fibers, giving them the appearance of ramen noodles (“ramen noodle sign”) (C).

Hypertrophic trabeculae, thin arachnoid-like membranes that normally suspend and support the nerve fibers within the CSF, can be found tethering the nerve fibers which can create multiple pockets of trapped CSF. A microdissection probe was used to open any blocked intrathecal cavities (Fig. 2).

The edges of the dural membrane were then imbricated and closed with 7-0 Prolene sutures to achieve a watertight seal. The closure was reinforced with a 3-layer patch using a highly purified collagen sponge (Duragen, Princeton, New Jersey) and fibrin sealant (Tisseal, Deerfield, Illinois).

After achieving hemostasis, the wound was closed in multiple layers, using a running, uninterrupted stitch to close the fascia and standard technique to close the dermis and skin. No drain was used.

Patients were allowed to ambulate immediately after surgery. All patients were discharged the same day. Patients were evaluated at routine intervals, typically at 1 week, 6 weeks, 3 months, 6 months, 12 months, and every year thereafter. All patients had at least 6 months follow-up.

## Results

### Surgical outcomes

A total of 19 patients met the surgical criteria, 68.4% female and 31.6% male, with a mean age of 45.5 ± 15.9 years (range 17.0–71.0). Patients with a minimum follow-up of 6 months were included in the analysis, with an average follow-up period of 18.2 ± 10.5 months (6.6–43.3).

The following findings during surgery were noteworthy. First, evidence of chronic inflammation was appreciated, marked by dense epidural adhesions along with increased vascular injection of the dural membrane adjacent to the cyst (Fig. 1). Upon opening the dural membrane, multiple small pockets of cysts were observed. Furthermore, some nerve rootlets displayed a tortuous course, resembling a packet of dry ramen noodles (Fig. 2, “ramen noodle sign”), similar to the appearance of the cauda equina in the lumbar spine cephalad to areas of severe stenosis.

Overall, 14 out of 19 patients (73.7%) reported clinical improvement after surgery based on the PGI-I scale, with an average PGI-I score of 2.6 ± 1.2 (range 1.0–4.0). Of these, 2 patients (10.5%) reported feeling “very much better” (PGI-I = 1), 9 patients (47.4%) reported feeling “much better” (PGI-I = 2), and 3 patients (15.8%) reported feeling “a little better” (PGI-I = 3).

Five patients (26.3%) did not experience any improvement (PGI-I = 4), while no patients reported worsening of symptoms (PGI-I = 5, 6, or 7). One patient who underwent surgery despite a negative diagnostic response (postinjection PGI-I = 4) had no improvement after surgery (postsurgery PGI-I = 4). In addition, 1 patient underwent surgery without a preoperative diagnostic injection and had improvement in symptoms after surgery (PGI-I = 3).

In the preoperative assessment, patients demonstrated an average ODI score of 32.9 ± 19.8 (range 0.0–70.0), which improved postoperatively to an average score of 28.4 ± 24.1 (range 0.0–72.0). Similarly, the SDS-R score decreased from a preoperative average of 30.9 ± 11.3 (range 8.0–52.0) to a postoperative average score of 26.8 ± 14.4 (range 0.0–45.0). The VAS for back pain also improved, with a preoperative average score of 4.8 ± 3.6 (range 0.0–10.0) decreasing to a postoperative average score of 3.2 ± 2.7 (range 0.0–9.0).

No patients reported a worsening of symptoms, and all were discharged on the day of surgery. There were no cases of CSF leaks, wound complications, or cyst recurrences at the most recent follow-up.

### Diagnostic injections

Eighteen of 19 patients received a diagnostic caudal steroid injection, with an average injection PGI-I score of 1.83 (range 1–4); 1 patient did not undergo a diagnostic injection. Seventeen of those 18 patients experienced a positive diagnostic injection response, whereas 1 patient had a negative diagnostic response.

The patient with a negative diagnostic injection response of PGI-I = 4 did not experience improvement after surgery, as the surgical PGI-I remained 4. The patient who did not undergo a diagnostic injection did show improvement after surgery, with a surgical PGI-I of 3.

Of the 17 patients who had a positive diagnostic response to their caudal epidural steroid injection with both anesthetic and steroid, the surgical success rate was 76.5% (13/17). As previously mentioned, the overall surgical success rate among all 19 patients was 73.7% (14/19).

### Statistical analysis

The Wilcoxon Signed-Rank test was used to evaluate changes across clinical outcome measures. Analyses were conducted for the SDS-R, ODI, and Visual Analog Scale for Back Pain (VAS Back).

Statistically significant improvement in SDS-R scores were observed from a preoperative score of 31.5 [IQR: 16] to 30 after surgery [IQR: 18] (*W* = 3, *Z* = −2.10, p < .05; *N* = 8). ODI scores showed a reduction from a median of 37 [IQR: 22] to 23 [IQR: 36], but the change was not statistically significant (*W* = 10, *Z* = −1.12, p > .05; *N* = 8). VAS Back pain scores decreased from a median of 6 [IQR: 7] to 3 [IQR: 4.5], but did not reach statistical significance (*W* = 19, *Z* = −1.57, p > .05; *N* = 12).

All analyses were performed using an online Wilcoxon Signed-Rank test calculator (SocSciStatistics.com). A 2-tailed p-value <.05 was considered statistically significant. Results are reported as medians with interquartile ranges (IQR) to reflect the nonparametric nature of the data.

## Discussion

Symptomatic Tarlov cysts are known to cause urinary dysfunction, as well as back and leg pain. Given the common neural pathways for urinary and sexual function [[7], [8], [9]], it is not surprising that patients with symptomatic Tarlov cysts can also develop symptoms of sexual dysfunction. This report describes a multidisciplinary approach to the evaluation and management of patients with sexual dysfunction due to symptomatic Tarlov cysts.

All patients had an initial evaluation by a sexual medicine expert to assess possible etiologies of sexual dysfunction. In patients with sexual dysfunction suspected of having neurologic etiologies, neurogenital testing is employed to further localize the pathology within 5 regions, with region 3 localized to the lumbosacral spine. Patients with no other identified causes of pelvic dysesthesia in whom neurogenital testing identifies region 3 as the primary abnormality, and who have Tarlov cysts detected on MRI, undergo diagnostic injections. Patients with a positive diagnostic response are offered surgery via Tarlov cyst excision and imbrication.

In a group of carefully selected patients, surgical treatment of symptomatic Tarlov cysts led to significant improvement in sexual dysfunction with a low complication rate. This is the largest series of patients with PGAD/GPD who underwent Tarlov cyst excision and imbrication to date, highlighting the relationship between sexual dysfunction and symptomatic Tarlov cysts.

Historically, the outcomes of Tarlov cyst surgery have been characterized by suboptimal results and a significant complication rate, ranging between 15% and 40% [5,13,14]. The low complication rate in the present report is likely related to the stage at which the diagnosis and surgical treatment occurred.

Most prior reports of Tarlov cyst surgery include patients with large Tarlov cysts presenting with complaints of urinary dysfunction. These large cysts often have significant bone erosion of the surrounding sacrum, which likely represents a late stage of disease. While the patients with pelvic dysesthesia in the present report had some urinary symptoms, none presented with urinary incontinence at the time of surgery.

Our multidisciplinary team, consisting of a urologist specialized in sexual medicine, a neurophysiologist, a sexual medicine researcher, and a spine surgeon, developed a strategy to identify patients with smaller cysts before the onset of irreversible symptoms such as urinary dysfunction. It is intriguing to consider that sexual dysfunction may precede urinary incontinence in the natural history of the disease.

Therefore, assessing patients with Tarlov cysts for sexual dysfunction, even in the case of small cysts, and assessing patients with sexual dysfunction for Tarlov cysts, may enable more successful surgical treatment.

It is unclear why some Tarlov cysts are symptomatic and others are not. Based on the diagnostic strategy, it seems likely that our patients suffered from a heightened state of inflammation. A previously asymptomatic Tarlov cyst may become symptomatic due to activation and persistence of inflammation, similar to other chronic inflammatory disorders such as lateral epicondylitis, rotator cuff tendinitis, and inflammatory bowel disease.

During Tarlov cyst surgery, we found gross evidence of chronic inflammation, with obvious epidural adhesions along with focal erythema of the dural membrane adjacent to the Tarlov cysts. Upon performing the durotomy, it was evident that many cysts were multichambered and that the path of the nerve fibers was abnormal, i.e., adherent to the dural wall and/or along a tortuous path (“ramen noodle sign”) (Fig. 2). This tortuous pattern was seen in the cauda equina, immediately cephalad to an area of lumbar stenosis in some patients.

We hypothesize that inflammation can produce thickening of the trabeculae, the thin arachnoid-like membranes that help suspend and support the individual nerve fibers within the cerebrospinal fluid-filled cavity of the Tarlov cyst. These structures are composed of connective tissue and resemble the arachnoid trabeculae found in the subarachnoid space of the central nervous system.

Prior studies have shown that thickening of arachnoid trabeculae can create a functional one‑way valve mechanism for CSF, promoting progressive cyst enlargement and nerve distortion [15]. This leads to progressive inflammation, ultimately resulting in a self-perpetuating cycle.

The resulting distortion can disrupt sacral sensory pathways, which play key roles in genital sensation, arousal, and pelvic autonomic function, making this mechanism highly relevant to PGAD/GPD symptomatology [16]. Chronic inflammatory processes—such as cytokine upregulation, perineural fibrosis, and neural sensitization—are also known contributors to persistent radiculopathy and may further explain why certain Tarlov cysts evolve from asymptomatic to symptomatic lesions [17].

These mechanisms align with our intraoperative findings: many cysts were multichambered, and nerve fibers frequently demonstrated an abnormal, tortuous course (“ramen noodle sign”), a pattern similarly observed in the cauda equina proximal to areas of severe lumbar stenosis. Tarlov cyst surgery aims to halt cyst growth, disrupt the cyst walls to eliminate the one-way valve system, and initiate a healing response in which the inflammatory process resolves.

Patients with GPD face an unusual challenge in diagnosis. They display a wider variety of symptoms than typical spine patients with back pain. The functions and movements within the pelvis are significantly more intricate and multifaceted compared to those of the legs and feet.

In addition to pain and numbness, alterations in sensation may lead to urinary urgency, changes in sexual arousal, alterations in orgasmic function, and even anhedonia, marked by a lack of pleasure during sexual activities. The spectrum of these sexual functions is wider than that of somatic functions such as ankle strength or skin sensation.

Furthermore, modest patients may be more reluctant to mention details of their sexual function and urination, which can make meaningful conversations with their health care provider difficult to initiate and explore.

Patients with Tarlov cysts have historically been difficult to treat because most cysts observed on imaging studies are asymptomatic, limiting the utility of radiographic imaging alone in determining symptom causality. Functional assessment using targeted diagnostic injections played a critical role in the evaluation process. Response to diagnostic caudal injection served as a practical diagnostic test to help identify patients in whom Tarlov cysts were likely contributing to GPD, supporting a functional rather than purely radiographic approach to patient selection. Cyst size and number did not correlate with the likelihood of a positive diagnostic response, underscoring the limitations of relying on morphologic characteristics alone when selecting patients for intervention.

Several limitations of this study should be acknowledged. First, the small sample size limits the ability to perform formal statistical analyses or identify predictive factors associated with surgical outcomes. Second, comparison between operative and nonoperative Tarlov cysts was not feasible, as most cysts detected on imaging are asymptomatic and do not undergo intervention. As such, radiographic distinctions between treated and untreated cysts remain difficult to define.

Future studies with larger patient cohorts may allow more robust differentiation between symptomatic and asymptomatic Tarlov cysts, and improve understanding of factors contributing to symptom generation. As experience with functional diagnostic strategies expands, it may become possible to better discriminate clinically relevant cysts without reliance on invasive testing, thereby refining diagnostic accuracy and treatment selection.

## Conclusion

Patients with GPD such as sexual dysfunction, pelvic pain, urinary urgency, or other pelvic symptoms not responding to traditional treatments should be assessed for the presence of symptomatic Tarlov cysts. These patients face significant functional limitations and a compromised quality of life. A multidisciplinary approach to evaluating and managing Tarlov cysts can lead to significant symptom relief with a low complication rate.

## Declaration of competing interest

Choll W. Kim is a consultant for Elliquence, Globus Medical.
